# **Which UHC? Features for Equity and Universalism** Comment on "Universal Health Coverage for Non-Communicable Diseases and Health Equity: Lessons From Australian Primary Healthcare"

**DOI:** 10.34172/ijhpm.2021.89

**Published:** 2021-08-02

**Authors:** Rene Loewenson

**Affiliations:** Training and Research Support Centre, Harare, Zimbabwe.

**Keywords:** Heath Policy, Health Systems, Primary Healthcare, Health Equity, Universal Health Coverage

## Abstract

Equity and universality are implicit in universal health coverage (UHC), although ambiguity has led to differing interpretations and policy emphases that limit their achievement. Diverse country experiences indicate a policy focus on differences in service availability and costs of care, and neoliberal policies that have focused UHC on segmented financing and disease-focused benefit packages, ignoring evidence on financing, service, rights-based and social features that enable equity, continuity of care and improved population health. Public policies that do not confront these neoliberal pressures limit equity-promoting features in UHC. In raising the impetus for UHC and widening public awareness of the need for public health systems, coronavirus disease 2019 (COVID-19) presents an opportunity for challenging market driven approaches to UHC, but also a need to make clear the features that are essential for ensuring equity in the progression towards universal health systems.

## Introduction

 The recent paper by Fisher et al^1^ in this journal raises useful lessons from Australia on the central role of primary healthcare (PHC) in universal health coverage (UHC), and on the policy measures needed to support equity and to manage common non-communicable diseases. As the authors note, the World Health Organization (WHO) defines UHC as when ‘all individuals and communities receive the health services they need without suffering financial hardship’ including ‘health promotion … prevention, treatment, rehabilitation, and palliative care’ (p. 2).^1^

 Universality has been a longstanding principle of health systems, although often not applied in practice. It was central to the 1978 Alma Ata declaration on PHC and to healthcare as a fundamental right in the 1966 International Covenant on Economic, Social, and Cultural Rights.^2,3^ It is implicit in UHC, although ambiguity has led in practice to differing interpretations and areas of policy emphasis to achieve it. For some it implies increasing revenues, whether this increases the segmentation of financing or not, and whether the services funded are public or private. For others, “any policy that fragments more than it unifies or results in segmented financing or pools of beneficiaries inherently goes against universalism, even if it is called universal”(p. 20). There are also different understandings of what services should be covered. The WHO definition of UHC and PHC clearly include health promotion, disease prevention, personal care, community engagement and public health action to address and regulate key social determinants of health (SDH). However, services funded by voluntary insurance and in resource constrained settings often focus on packages of facility-based curative services.^4^ While this may respond to financial considerations, disease-based and “silo’ed” curative models undermine the management of co-morbidities and measures for population health gains, particularly for disadvantaged communities, weakening the very universality and financial protection aimed for.^5^ As Fisher et al^1^ argue, we need to move beyond terms and labels to critically understand the implications of the different policy measures being promoted within UHC.

 In the context of a coronavirus disease 2019 (COVID-19) pandemic that has raised an impetus for UHC, this commentary explores further the implications of the choices made of policy measures for UHC, particularly for how far they advance equity.

## Equity Is Not Inevitable in Advancing UHC

 Equity in terms of access to services in relation to heath need rather than ability to pay, and closing avoidable and unfair social inequalities in health outcomes seems inherent to UHC as a goal. It is, however, not an inevitable consequence of how UHC is advanced. It results not only from the content and technical approaches, but also from how far social justice, rights, solidarity and the agency of those with greatest health need are embedded within what is prioritised, reflecting political economies and public values within and across countries.^6,7^

 Equity in UHC (EUHC) opens different lenses. From an economic lens, financial protection against impoverishment and equity in resource allocation demand overcoming fragmentation in funding pools, to enable income and risk cross-subsidies and to avoid two tier systems. For EUHC this needs to be an early measure, as politically and technically it is often difficult to merge pools at later stages.^7^ As we have witnessed so sharply in the COVID-19 pandemic, EUHC also implies overcoming market barriers to the distributed production of, supply chains for, and access to diagnostics, therapeutics, vaccines and other essential health commodities.^8^ From a social justice and public health lens, evidence, including from the 2008 WHO Commission on SDH, has showed that EUHC demands comprehensive approaches, aligned across different sectors, to address the multiple health needs of disadvantaged communities. Further, it demands ‘upstream’ action on the deeper determinants of social inequalities in health. While these actions may lie beyond the health sector, they call for public health authority and leadership to embed health equity within other sector policies, laws and measures and to regulate market practice.^1,2^ From a lens of human rights and social power, EUHC implies redressing significant imbalances in the entitlements, capabilities, agency and collective power different groups have to claim rights to health, to direct resources to meet their health needs, and to co-determine actions that affect them. It also implies ensuring the rules, capabilities and orientation of institutions and personnel to support this.^6,7^

 Behind policy statements on UHC therefore, the policy content, interests and practice need to be critically analysed for which of these dimensions of equity are being promoted, and which ignored.

 An analysis of UHC policies in Benin and Senegal, for example, found that while inequalities were recognised and equity referred to as a guiding principle, the focus was largely on differences in service availability and costs of care. There was limited consideration of other dimensions of equity, or of remedial measures to address the differentials found.^6^

 In many countries, UHC-related initiatives expand domestic revenue through different forms of voluntary insurance, despite unequivocal advice from WHO and others that this is not a feasible approach to achieving UHC. Universality calls for mandatory pre-payment through improved tax collection, particularly from high net-worth individuals and multinational corporations, or mandatory progressive national health insurance.^7^ The experience Fisher et al^1^describe in Australia validates cautions around hoping to address fragmented financing after establishing segregated schemes. Despite it being a ‘mature’ UHC system, they found that segmented public and private funding persisted, with a focus on funding of episodic care and underinvestment in the public health measures tackling corporate practices and upstream determinants. These features were observed to contribute to inequities in health, and were attributed to the dominant neoliberal political values and interests shaping and influencing UHC-related policies.

 Globalisation has spread such neoliberal political values, policies and interests across many countries. Reforms driven by Organisation for Economic Co-operation and Development country governments and Bretton-Woods institutions favoured free trade, deregulated markets and reduced social budgets, promoting ‘healthy’ neoliberal economies’ as a priority over and a means to achieving healthy people.^2^ Policy commitments to UHC were thus driven in part by pressures over the evident underfunding of health systems and rising public health risks after decades of neoliberal economic policies, as countries made efforts to reintroduce universal systems.^2^ While efficiency, privatisation and deregulation goals in a dominant neoliberal paradigm have affected countries at all income levels, in low and middle income countries they led to a shift towards minimum packages of biomedical interventions for high impact diseases, chosen for their cost effectiveness and combined with target-driven funding to promote efficiencies.^2,3^ Backed by significant international resources for selective, vertical disease programmes, this did lead to coverage gains in the biomedical services targeted.^2^ It has, however, often excluded chronic conditions, public health infrastructures and regulation of market practices; and has poorly recognised the increased investments needed to reach and encourage uptake in more marginalised groups as well as the wider social return on investment approaches needed to track investments against such outcomes.^2-4^

 Neoliberal models would appear to have cut back on the very areas of investment that evidence suggests have enabled equity, continuity of care, improved population health, and indeed value for money in health.^1-3^ This includes investment in enrolment of populations with local services, in community outreach and comprehensive PHC, in community health workers and integrated multidisciplinary health and social care teams, and in focusing capacities in public sector frontline services, and in providing incentives for primary care to provide effective first facility contact and co-ordination of referral.^5^ A focus on specific diseases detracted from person-centred, area-based and population approaches that enable PHC as an entry point to link clients with the other services and programmes needed to improve their health, as for example is implemented in Chile’s public sector biopsychosocial approach.^5,9^ Politically, minimum health packages and target-driven funding sent a message that services would be covered on the basis of what was affordable within the parameters set by a neoliberal economy, rather than what should be delivered as a collective, social right based on need, making some states cautious about the implications of including rights to healthcare in their constitutions.^3,4^

 Involving communities and affected stakeholders in monitoring of services and achievement of targets promotes local accountability. However, this is a significantly more limited vision of capabilities, agency and power than is envisaged, or indeed needed for equity and universalism, particularly given the different socio-political, economic and technical ideas and interests that shape and influence health policy. For example, in participatory research with primary care level health workers, community and health civil society members from seven sites in five countries in east and southern Africa, target-driven approaches introduced to improve coverage and remove fee charges for selected facility-based services included service monitoring by local committees. While the participants noted improved coverage in the areas targeted, they perceived that they had limited flexibility in choosing what was prioritised. They felt that if they had more say, they would fund chronic conditions, community level prevention, community health worker roles, and social challenges such as gender-based violence. These were areas that were underfunded or even ignored, despite their perceived importance for equity.^10^In contrast, providing flexibilities for and measures to support and exchange learning from local systems has supported innovations for equity. One example of this was Chile’s ‘Colaboración Publica Salud’ platform, that provided a digital space for local health system actors to share challenges and how they addressed them.^9^

 These experiences in different settings are consistent with findings of desk reviews of UHC programmes across a range of countries. In these reviews a dominant focus on increasing coverage and reducing cost barriers to access for the most disadvantaged groups, including through targeted funding, while relevant, was observed to have left significant gaps in other key strategies for EUHC. Such strategies include equitable allocation of resources, pooling for cross subsidies, providing holistic services, and ensuring social agency and fair procedure in institutional governance.^6,11^ Public sectors that do not confront neoliberal economic policy principles and the international and domestic lobbies promoting them in advancing UHC are argued to self-limit equity-promoting options, particularly those that promote comprehensive public health and PHC, market regulation, human rights and social power.^6^

## COVID-19 Exposing Tensions and Catalysing Review

 It is difficult to comment on whether public sectors will build a more robust confrontation with neoliberal policies and interests undermining EUHC without considering how COVID-19 may affect this. Across countries, responses to the pandemic reflect the same long-standing tensions between comprehensive, participatory responses and a primarily biomedical, behavioural focused response. Comprehensive approaches include not only biomedical measures, but also action on environmental and working conditions, food security, psychosocial and social protection. A biomedical, behavioural response to the pandemic has often manifested in ‘command-and-control’ and even militarised approaches, with limited consultation of those affected. Such rapid, over-centralised biomedical approaches come at the cost of harm to relationships between communities and the state that are essential for public health, including for vaccine uptake, and for action on SDH such as gender violence.^12^

 Yet the pandemic has certainly provided a rupture in complacency. There have been new calls for global public goods, challenges to patent rights, demands for reinvestment in health and social systems and rethinking on political and economic measures for collective security within and across countries. The pandemic has exacerbated and exposed corruption around public funds in the response, and the negative consequences of underfunding public health systems.^12-14^ It has exposed inequalities within and across countries that heighten risk and vulnerability, as well as powerful interests that have self-protected, further widening inequality and prejudice, triggering calls for explicit attention to equity.^12,13^

 As Arundathi Roy observed, pandemics offer a chance to break with the past, and “nothing could be worse than a return to normality.”^13^ For example, during the 2014-2016 Ebola crisis in Liberia, Redemption Hospital, Monrovia’s only free-of-charge public hospital closed its inpatient services for six months, leaving local women without maternity care. The loss of community trust in the service led health and hospital workers, community members, community birth attendants and local leaders to confront the negative staff attitudes, poor links with community and primary care services and a lack of referral networks between different parts of the system that undermined services before, during and after the pandemic. Their review led to shared proposals for a more comprehensive approach to maternal health.^15^

 So will COVID-19 provide muscle to public sector, health and social actors beyond the pressures of the immediate response to the pandemic, to provide a more robust confrontation with neoliberal policies, values and interests that have influenced and undermined equity and universality in health?

 The difficulties that many states faced to provide an adequate and comprehensive response to COVID-19 are argued to have strengthened the impetus for UHC. However, if this is the UHC that sustains the same narrow lens on reactive technocratic, biomedical and individual behavioural approaches, it is argued that the pandemic may deepen the financialisation and inequity in health systems.^14^ Lopez Cabello^14^ notes that the pandemic has widened public awareness of the critical role that universal, equitable public health systems play in our lives. We are thus in an important moment to challenge neoliberal, market-driven approaches to UHC, and to make clear the features that are essential for us to advance towards equitable, universal health systems.

## Ethical issues

 Not applicable.

## Competing interests

 Author declares that she has no competing interests.

## Author’s contribution

 RL is the single author of the paper.
